# Correction to “Anthropometric Failure and Associated Factors Among 6–59 Month Children Living in Gicumbi District”

**DOI:** 10.1002/puh2.70196

**Published:** 2026-03-07

Y. D. Umwungerimwiza, P. Kwizera, E. Matsiko, et al., “Anthropometric Failure and Associated Factors Among 6–59 Month Children Living in Gicumbi District” *Public Health Challenges* 5 (2026): e70183, https://onlinelibrary.wiley.com/doi/10.1002/puh2.70183.

Following the publication of the above‐mentioned article, errors were identified in the presentation of some authors’ names. The correct author names are provided below:

- “Alphonsine Nyirahabimana” to be corrected to “Alphonsine Nyirahabineza.”
- “Phiona Nziza” to be corrected to “Phionah Nziza.”
- “Ilinde Niyigena” to be corrected to “Niyigena Delice Ilinde.”
- “Sunday François Sunday” to be corrected to “François Xavier Sunday.”

These corrections relate solely to the spelling and order of authors’ names and do not affect the scientific content, analysis, or conclusions of the article.

The online version has been corrected.

We apologize for these errors.

